# A multi-tissue genome-scale metabolic modeling framework for the analysis of whole plant systems

**DOI:** 10.3389/fpls.2015.00004

**Published:** 2015-01-22

**Authors:** Cristiana Gomes de Oliveira Dal'Molin, Lake-Ee Quek, Pedro A. Saa, Lars K. Nielsen

**Affiliations:** Centre for Systems and Synthetic Biology, Australian Institute for Bioengineering and Nanotechnology, The University of QueenslandBrisbane, Qld, Australia

**Keywords:** multi-tissue, genome-scale, modeling, plant metabolism, AraGEM

## Abstract

Genome scale metabolic modeling has traditionally been used to explore metabolism of individual cells or tissues. In higher organisms, the metabolism of individual tissues and organs is coordinated for the overall growth and well-being of the organism. Understanding the dependencies and rationale for multicellular metabolism is far from trivial. Here, we have advanced the use of AraGEM (a genome-scale reconstruction of Arabidopsis metabolism) in a multi-tissue context to understand how plants grow utilizing their leaf, stem and root systems across the day-night (diurnal) cycle. Six tissue compartments were created, each with their own distinct set of metabolic capabilities, and hence a reliance on other compartments for support. We used the multi-tissue framework to explore differences in the “division-of-labor” between the sources and sink tissues in response to: (a) the energy demand for the translocation of C and N species in between tissues; and (b) the use of two distinct nitrogen sources (NO^−^_3_ or NH^+^_4_). The “division-of-labor” between compartments was investigated using a minimum energy (photon) objective function. Random sampling of the solution space was used to explore the flux distributions under different scenarios as well as to identify highly coupled reaction sets in different tissues and organelles. Efficient identification of these sets was achieved by casting this problem as a maximum clique enumeration problem. The framework also enabled assessing the impact of energetic constraints in resource (redox and ATP) allocation between leaf, stem, and root tissues required for efficient carbon and nitrogen assimilation, including the diurnal cycle constraint forcing the plant to set aside resources during the day and defer metabolic processes that are more efficiently performed at night. This study is a first step toward autonomous modeling of whole plant metabolism.

## Introduction

**Figure 1:**
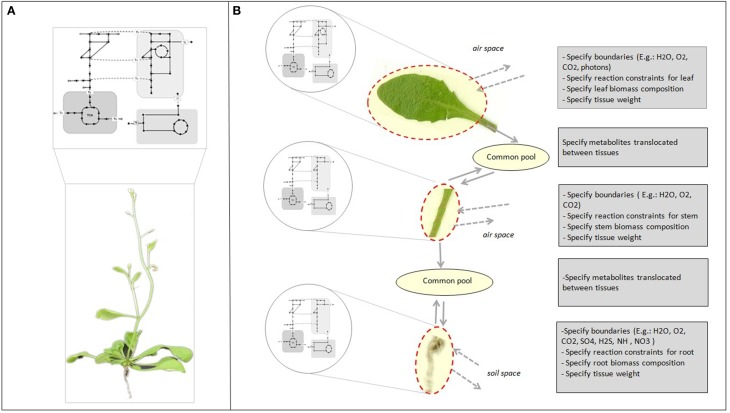
**Genome-scale metabolic reconstruction and specific tissue models. (A)** The reconstruction represents the full set of metabolic reactions of the organism. **(B)** Tissue or cell specific models can be derived from the metabolic reconstruction to represent tissue and cell specific functions by adding physical–chemical constraints and tissue biomass compositional data. The common pools enable translocation of metabolites between tissues. Chemical species that are translocated between tissues are internal metabolites and must be balanced.

Genome-scale reconstruction and modeling of metabolism enables the integration of knowledge at different levels of the cascade from genes over proteins to metabolic fluxes. This is pivotal to develop an understanding of how individual components in a system interact and influence overall cell function (Oberhardt et al., 2009; Blazeck and Alper, 2010). Reconstructions capture our current knowledge of the full set of metabolic capabilities of an organism (Figure 1A). In multicellular organisms, specific tissues only utilize a subset of the full set of capabilities encoded by the genome, and at the same time depend on other tissues for support. While it is relatively easy to generate cell or tissue specific models from single genome reconstructions (Figure 1B) (Bordbar et al., 2011; de Oliveira Dal'Molin and Nielsen, 2013), the real challenge is to gain insight into the intricate interactions between the various tissue types and unravel their core metabolic dependencies.

In the past few years, genome-scale reconstructions were developed for several plant species, including *Arabidopsis thaliana* (Poolman et al., 2009; de Oliveira Dal'Molin et al., 2010a; Mintz-Oron et al., 2012), maize (*Zea mays*) (de Oliveira Dal'Molin et al., 2010b; Saha et al., 2011), sorghum (*Sorghum bi-color*), and sugarcane (*Saccharum officinarum*) (de Oliveira Dal'Molin et al., 2010b). Our Arabidopsis genome-scale reconstruction (AraGEM 1.0) was developed to provide a global genome-scale description of plant metabolic capabilities (Figure 1A). While AraGEM does not contain tissue-specific information, it can be used to model important metabolic scenarios of both photosynthetic and non-photosynthetic tissues (de Oliveira Dal'Molin et al., 2010a). In separate work, Mintz-Oron et al. developed a computational pipeline for cell compartmentalization and generated 10 individual tissue-specific models, which cover primary and some secondary pathways of Arabidopsis (Mintz-Oron et al., 2012). More recently, Arnold et al. has proposed an Arabidopsis core reconstruction and its utility to estimate the metabolic costs of enzyme production (Arnold and Nikoloski, 2013).

The Arabidopsis reconstructions have been explored using several constraint-based modeling approaches and strong correlations have been observed between predicted and observed results (Poolman et al., 2009; de Oliveira Dal'Molin et al., 2010a,b, 2014; Saha et al., 2011). Multi-tissue analysis has so far not been performed directly on Arabidopsis models. However, AraGEM formed the basis of our C4 model (C4GEM), which was used to model C4 photosynthesis considering the tissue–tissue interaction between mesophyll and bundle sheath cells (de Oliveira Dal'Molin et al., 2010b). Two instances of the model, one for bundle sheath and one for mesophyll, were connected through exchange of metabolites via plasmodesmata. C4GEM predicted the classical C4 photosynthesis pathway and was used to: (i) investigate the effect in organelle function in mesophyll and bundle sheath, (ii) explore the metabolic activities around photosystem I and photosystem II for three different C4 subtypes, and (iii) to explore the effects of CO_2_ leakage out of bundle sheath.

Ultimately, the goal is to advance the reconstruction of metabolism at the whole-plant level. Ideally, whole plant models would be used to obtain non-intuitive results from simple, observable multi-tissue constraints. One of many potential uses of the whole plant framework is to guide genetic engineering strategies to improve the nitrogen and carbon use efficiency of crop plants. Physiological representation and overall analysis, however, cannot be achieved unless an integrated multi-tissue modeling framework for plants is developed. Here we have advanced the use of AraGEM (de Oliveira Dal'Molin et al., 2010a) in a multi-tissue context to explore complex interactions of carbon, nitrogen metabolism, and resource allocation in the whole plant across the diurnal cycle. Using resource utilization as the optimality criterion, we explore how plants efficiently allocate resources between leaf, stem, and root while performing the necessary carbon and nitrogen translocation over the diurnal cycle.

## Materials and methods

### Multi-tissue framework

Conventional genome-scale modeling deals with a cellular network that exchange metabolites directly with the surrounding. We recently demonstrated that the availability of these exchanges can be controlled in a context-dependent manner (Quek et al., 2014).

When modeling whole plant systems, we must consider the tissues, external pool (boundaries exchanges), exchange of metabolites between tissues (common pools), temporal storage and retrieval of metabolites and in particular, the diurnal cycle constraint.

The multi-tissue modeling framework developed here differs from the approaches used in modeling microbiome (Mahadevan and Henson, 2012) and multi-tissues of human (Bordbar et al., 2011). Although these problems can be solved by using constraint-based optimization, the objective function, interaction pools, boundaries and model constraints are different. When modeling microbiomes for example, it is important to consider the metabolic interactions that are possible in the studied community (i.e., competition, cross-feeding, syntrophy or mutualism). In most studies, the community objective has been assumed to be growth rate maximization of the individual microbes, but a community level objective function in addition to the individual species objective function of growth rate maximization has been also considered (Zomorrodi and Maranas, 2012). The individual tissues and organs of plants do not interact by competition. Instead, the metabolism of individual tissues and organs is coordinated for the overall growth and well-being of the organism. Here we tested the framework under the assumption that the whole plant metabolic network will minimize energy usage (photon capture) for plant growth (see Model Assumptions and Constraints Section).

#### Common pool

**Figure 2:**
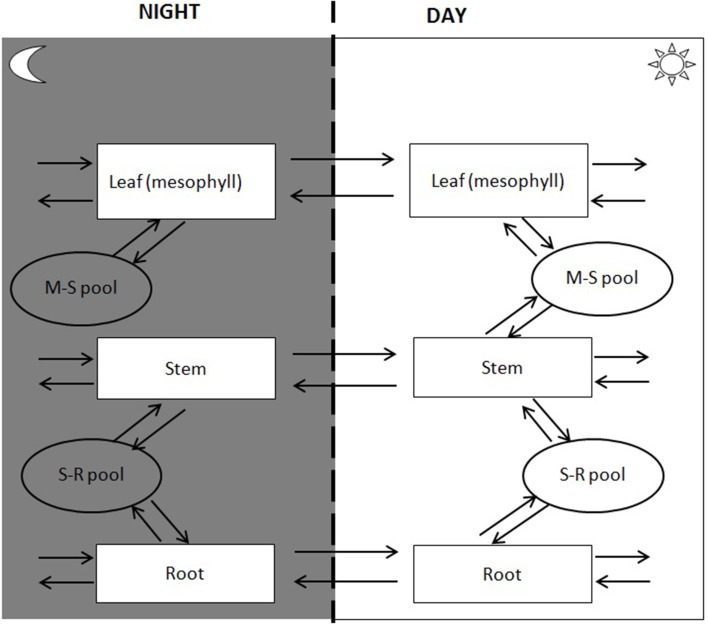
**Tissue compartments, intercellular translocation and common pool over day-night period**.

Spatial transport is captured by defining a shared resource pool or “common pool” (CP). CP has no storage capacity, so transport to the pool from one tissue must be matched by transport to other tissues. A model may have several CPs to describe distinct pools shared by some but not all tissue types. A simple C4 model, for example, would have a CP describing exchange between bundle sheath and mesophyll tissue as well as a CP describing translocation through the vasculature. Transport mechanisms fall into two catagories passive and active. Passive transport mechanisms do not require the cell to do work for the substance to enter or leave the cell (e.g., diffusion or water transport). Active transport mechanisms involve the cell to use cellular energy usually in the form of ATP. In the multi-tissue model, active transport is captured by coupling transport to ATP hydrolysis. The difference in energetics of loading and unloading can be captured by introducing separate transport for export and import. For the purpose of illustration, the current study will consider a three-tissue (root, stem, and leaf tissues) model with two interstitial CPs, one for leaf-stem exchange and one for stem-root exchange (Figure 2).

#### Storage pool

Temporal storage and retrieval of metabolites is managed through the introduction of a storage pool (SP). We can divide time into as few or many periods as required. The key assumption is that there is no net accumulation across all periods, i.e., whatever is stored in one period must be retrieved in the other periods. For illustration purposes, we will here consider a day-night cycle with leaf starch being the only stored compound.

#### Stoichiometric matrix

We extended the concept of stoichiometric modeling to describe the metabolite balance constraints for the whole-plant system. Using an existing genome-scale model, we can define an internal stoichiometric matrix *S* (i.e., excluding all transporters) and three types of transport matrices. Matrix *E* represents the exchange reactions of metabolites in *S* with the environment, matrix *T* the transport reactions of metabolites in *S* with the common pool *CP*, and matrix *A* the accumulation reactions of metabolites in *S* into a temporal storage pool *SP*. The number of rows in matrix *CP* or *SP* depends on the number of metabolites shared or stored, respectively.

The stoichiometric matrix block representing the leaf, stem and root tissues (*l*, *s*, *r*) exchanging with the environment and common pools for a given period is shown below:

(1) $$s_{block}\text{​}=\text{​​}\left[\begin{array}{cccccccccc} S_{l} & 0 & 0 & E_{l\to } & 0 & 0 & T_{l\to s} & 0 & 0 & 0\\ 0 & S_{s} & 0 & 0 & E_{s\to } & 0 & 0 & T_{s\to l} & T_{s\to r} & 0\\ 0 & 0 & S_{r} & 0 & 0 & E_{r\to } & 0 & 0 & 0 & T_{r\to s}\\ 0 & 0 & 0 & 0 & 0 & 0 & \omega_{l}CP_{ls} & \omega_{s}CP_{ls} & 0 & 0\\ 0 & 0 & 0 & 0 & 0 & 0 & 0 & 0 & \omega_{s}CP_{sr} & \omega_{r}CP_{sr} \end{array}\text{​​​​}\right]$$

Mass fractions ω_*l*_, ω_*s*_, and ω_*r*_ for the different three tissue types (leaf, stem and roots) were introduced to convert intrinsic tissue fluxes (moles/g DW/h) to extrinsic fluxes (moles/h), in order to balance fluxes between tissues.

Temporal separation (i.e., day-night cycle) was achieved by duplicating compartments for both tissues and common pools and introducing SP. The large stoichiometric matrix linking resource storage between day-night cycle is:

(2) $$S_{\text{whole plant}}=\left[\begin{array}{cccc} S_{block,day} & 0 & A_{day\to } & 0\\ 0 & S_{block,night} & 0 & A_{night\to }\\ 0 & 0 & \omega_{d}SP & \omega_{n}SP \end{array}\right]$$

Where time fractions ω_*d*_, ω_*n*_ were introduced to represent the fraction of hours day light is available to the plant, in order to convert rates to total flows. The introduced mass and time fractions are shown in Supplemental Material (multi-tissue framework folder, read me file).

Flux balance analysis (FBA) (Orth et al., 2010) was used to investigate the uptake, assimilation and tissue reallocation of nitrogen and carbon. The above metabolic problem can be solved by linear programming using an objective function *f* and a set of flux boundary constraints (*v*_*lb*_, *v*_*ub*_).

(3) $$\begin{array}{l} \min\;f^{\text{T}}v\\ S_{\text{whole plant}}\cdot v=0\\ v_{lb}\leq v\leq v_{ub} \end{array}$$

### Model assumptions and constraints

Equations (1)–(3) define a generic FBA model for a given genome scale stoichiometry with a defined set of tissues and pools. Specific models are formulated by specifying the objective function weights, *f*, the exchange weights, *w*, and the flux through a subset of reactions. In the current example, we will consider a minimal specification of fluxes to explore flux coupling and photon optimal flux distribution assuming the plant has access to the full set of reactions. In alternative formulations, transcriptomics and proteomics may be used to define tissue specific reactions based on the gene-protein-reaction mapping.

#### Metabolic objective

We assume that plant metabolism (e.g., kinetics and regulatory factors) has evolved to become efficient at utilizing photons for growth (Equation 3). Efficient resource usage has been successfully applied as the metabolic objective to predict single and two-tissue metabolic function (de Oliveira Dal'Molin et al., 2010a,b). Efficient photon utilization was used for the current model objective and implemented as photon minimization subject to achieving a given rate of biomass synthesis.

Plant growth rate and biomass composition for each tissue were estimated based on literature data (Pooter and Bergkotte, 1992) (see Supplemental Material, Tables S1, S2). These numbers were used to define plant growth rate and biomass composition for each tissue. The set of constraints (upper and lower boundaries) is shown in Supplemental Material (Table S2). At this stage, we do not account for nitrogen and carbon re-mobilization from senescent leaves.

#### Phototrophic vs. heterotrophic

The leaf is treated as a phototrophic tissue, whereas the stem and root are treated as heterotrophic tissue. These tissue-specific activities were imposed using constraints on RuBisCO activity in root and stem, *v*^*RuBisCO*^_*root*_ = 0 and *v*^*RubisCO*^_*stem*_ = 0.

#### Diurnal cycle: starch links day/night period

Starch is the major form in which carbon is stored in plants. We assume a 12-h/12-h dark/light period and continual growth during the night. Thus, starch must accumulate during the day to meet metabolic requirements during the night, *v*^*starch biomass*^_*leaf, day*_ ≥ 0 and *v*^*starch biomass*^_*leaf, night*_ ≤ 0 (Smith et al., 2004).

#### Tissue translocation

The tissue model defines both the tissues and the metabolites pools used for interaction (Figure 1B). The boundaries are defined for each tissue and species defined for translocation (e.g., sucrose, amino acids, NO_3_, H_2_O) considering both day and night period. Phloem loading and unloading of amino acids and sucrose is dependent on an energy-requiring mechanism (Giaquinta, 1979; Servaites et al., 1979). However, identifying transport mechanisms responsible for translocation of photoassimilate to and from the leaf as well as to measure the energy demand for the translocation process has proven challenging. The multi-tissue framework incorporates translocation energetics through a “penalty weight” (*pw*) for the species translocated between tissues (captured by coupling transport to ATP hydrolysis). For example, the penalty weight for sucrose translocation can be described as:

$$\begin{array}{l} (pw)\left[\text{ATP + }{\text{H}}_{2}\text{O}\right]\text{ + }{\left[\text{Sucrose}\right]}_{\left(\text{leaf}\right)}\\ \;=(pw){\left[\text{ADP + Orthophosphate}\right]}_{\left(\text{leaf}\right)}\text{+ }{\text{Sucrose}}_{\left(\text{common pool}\right)} \end{array}$$

where *pw*^*sucrose*^_*leaf, CP*_ > 0, when the translocation process of sucrose from leaf to the common pool is considered active. Passive diffusion is described by defining zero penalty, e.g., *pw*^*sucrose*^_*leaf, CP*_ = 0.

By varying *pw*, we were able to evaluate the main changes in the metabolic network caused by an active translocation process compared to a passive translocation process for glutamate, nitrate and sucrose between tissues.

#### Nitrogen source

The model was used to contrast two nitrogen sources: nitrate and ammonium. The contrast was implemented by constraining uptake of the alternate nitrogen source, e.g., ammonium uptake was constrained to zero, *v*^*NH3 uptake*^_*root*_ = 0, to describe nitrate metabolism.

### Model analysis

#### Comparison of flux distributions under different conditions

The solution to any genome scale FBA problem (Equation 3) is degenerate, i.e., multiple solutions produce the same optimal value. Comparisons can be made using representative values determined by adding an additional criterion, for example, choose the optimal solution that minimizes total enzyme effort required. This can be calculated as an optimal solution to Equation (3) that also minimizes the L_1_-norm (taxicab norm):

(4) $$\begin{array}{l} \text{min }\sum_{i}\left|v_{i}\right|\\ \text{s}.\text{t}.\\ S_{\text{whole plant}}\cdot \;v=0\\ f^{\text{T}}v=Z^{\text{opt}}\\ v_{lb}\leq v\leq v_{ub} \end{array}$$

where *Z*^opt^ denotes the solution found in Equation (3).

#### Flux variability analysis

If no additional criterion is introduced, Flux Variability Analysis (FVA) (Mahadevan and Schilling, 2003) can be employed to determine which flux variables are fixed and which can vary under optimal conditions. FVA determines the lower and upper value for each flux, one at a time

(5) $$\begin{array}{l} \text{min/max }v_{i}\\ \text{s}.\text{t}.\\ S_{\text{whole plant}}\cdot \;v=0\\ f^{\text{T}}v=Z^{\text{opt}}\\ v_{lb}\leq v\leq v_{ub} \end{array}.$$

#### Random sampling of the solution space

FVA identifies the extreme values for each individual reaction. Uniform sampling of the solution space has been increasingly used for studying the correlation structure of metabolic networks (Almaas et al., 2004), unraveling transcriptional regulation in key enzymes (Price et al., 2004; Bordel et al., 2010) and determining important metabolic interactions between different cell types (Bordbar et al., 2011; Shoaie et al., 2013). In our case, we employed sampling to explore flux distributions under different scenarios as well as to identify coupled reaction sets in different tissues and organelles. Prior to sampling, we used FVA to remove all blocked reactions in the model given the constraints of the different scenarios analyzed. Next Monte Carlo sampling was performed using a modified version of the Artificially Centered Hit-and-Run (ACHR) algorithm (Kaufman and Smith, 1998), which is readily available within the COnstrained Based Reconstruction and Analysis (COBRA) Toolbox (Schellenberger et al., 2011). A total of 10^5^ uniformly distributed samples were generated and used to calculate pairwise. Pearson correlation coefficient (ρ) was calculated.

Modules of coupled reactions were identified from the sets of highly correlated reactions by casting the problem as the maximum clique enumeration problem (Eblen et al., 2012).

The adjacency matrix (*Adj*) was constructed based on the reactions (*i, j*) with correlations higher than a correlation cut-off (|ρ_*ij*_| > ρ_cut−off_). This matrix represents an undirected graph *G* = (*V*, *E*) consisting of a finite sets of vertices *V* (reactions) and a finite set of edges *E* (coupled reaction pairs). The problem of finding the largest sets of fully connected reaction modules can now be cast as listing maximal cliques. A clique is a subset *C* of the vertex set (*C* ⊆ *V*) such that every vertex in *C* is connected to all the other vertices in the clique, i.e., *C* is a complete graph. All maximal cliques were enumerated using the Bron–Kerbosch algorithm based on recursive backtracking (Bron and Kerbosch, 1973). In this way, we were able to identify and visualize maximal cliques involving highly correlated reactions from different tissues, organelles and day periods under different conditions.

#### Computational implementation

AraGEM was used as the base metabolic model for the construction of the multi-tissue model. FBA, FVA and coupling calculations were performed using Gurobi Optimizer 5.6 (Gurobi Optimization, Inc.) within the MATLAB 2013a environment (The MathWorks, Natick, MA). MATLAB scripts, along with instructions to use the scripts, are provided in Supplementary Materials.

## Results and discussion

Nitrogen use by plants involves uptake, assimilation and translocation/remobilization. Nitrogen is most often taken up by plants as water soluble nitrate (NO^−^_3_; usually the most abundant form), ammonium (NH^+^_4_) and to a lesser extent, as proteins or amino acids (Masclaux-Daubresse et al., 2010). The combined use of ammonium (NH^+^_4_)-based fertilizers and nitrification inhibitors can effectively alleviate the two main environmental problems associated with nitrogen fertilization, namely water pollution caused by nitrate leaching and gaseous emissions of nitrogenous compounds. As such, the use of NH^+^_4_ has been proposed as a good alternative to nitrate-based fertilizers (Lesschen et al., 2011). Once nitrogen has been taken up and assimilated, it is transported throughout the plant as glutamine, asparagine, glutamate, aspartate, NO^−^_3_ and NH^+^_4_ for utilization, storage and remobilization (McAllister et al., 2012). The assimilated nitrogen forms are transported via xylem and distributed to mesophyll cells, where they are either stored or utilized for carbon assimilation.

The current framework is flexible and allows for translocation between tissues and temporal storage of any number of components. For the sake of simplicity, we here use the simplest possible model based on the following assumptions: (i) most of C and N species are translocated in between tissues in the form of NO^−^_3_, glutamate, and sucrose; (ii) most of NH^+^_4_ is assimilated in roots rather than translocated to source tissues; (iii) starch is the major storage component and is accumulated in leaves; (iv) growth is constant day and night; and (v) the whole plant will use the minimum energy capture (i.e., best network performance with minimum photon usage).

Under these assumptions, we used the multi-tissue framework to explore differences in the “division-of-labor” between the source and sink tissues as a function of (a) the energy demand for the translocation of C and N species in between tissues, and (b) the nitrogen source (NO^−^_3_ or NH^+^_4_) used. The model-highlights discussed here are to show the potential use of the framework in whole-plant systems only. Biological interpretation requires a more detailed model as well as experimental validation.

### The effect of active transport on tissue-translocation in the network

**Figure 3:**
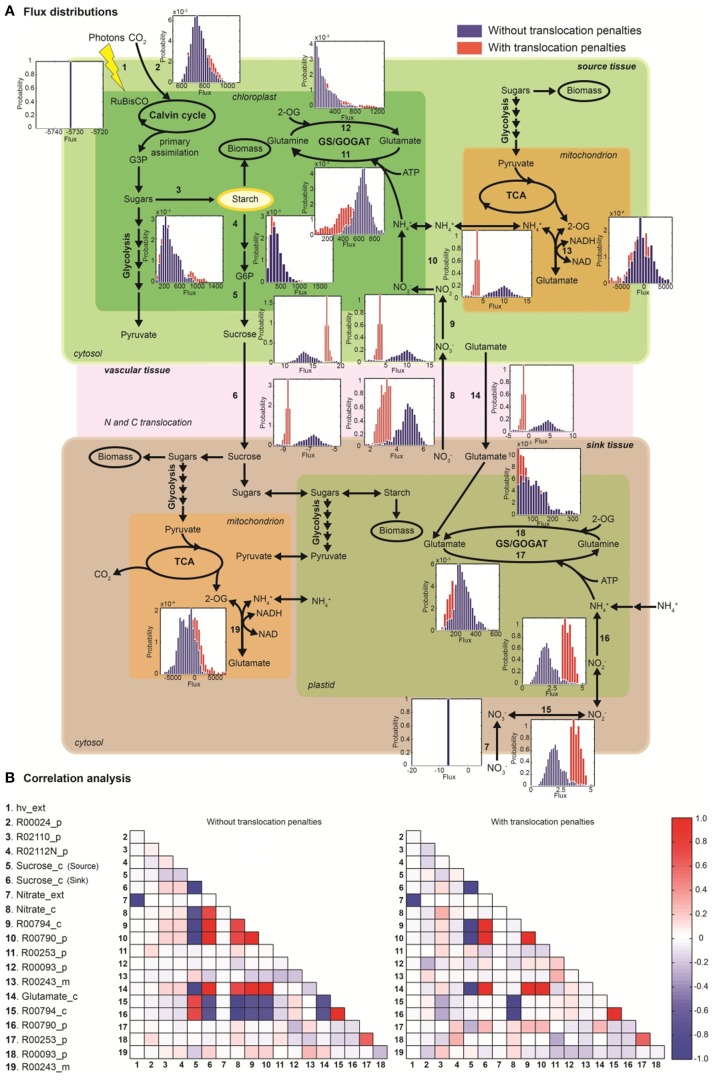
**The effect of active tissue translocation on nitrogen uptake and assimilation pathways using nitrate as nitrogen source. (A)** Flux distributions for the nitrogen uptake and assimilation pathways in the multi-tissue model. The histograms next to each reaction step represent the flux distributions with active tissue translocation (with translocation penalties, red) and with passive transport (without translocation penalties, blue) for sucrose, glutamate and nitrate species. Distributions shown are based on 10^5^ uniform samples from the solution space. **(B)** Correlation between fluxes were calculated between pairs of reactions of the multi-tissue model using the 10^5^ random sample points. Perfect positive and negative correlation (1.0, −1.0) are shown in dark red and blue, respectively.

Random sampling of the solution space was used to compare active (with energetic penalties) vs. passive (without penalties) tissue translocation under nitrate as sole N source (Figure 3). The model highlights that the restriction of one metabolic step, such as the increase in energy requirement for C or N species to be translocated between tissues, can have a profound effect on the behavior of the plant network as a whole. Figure 3A illustrates N uptake and assimilation pathways across sink and source tissues. The initial reduction of NO^−^_3_ to NO^−^_2_ occurs in the cytoplasm and it is carried out by nitrate reductase (step 9 and 15). Further reduction of NO^−^_2_ occurs in the plastid/chloroplast by nitrite reductase, which converts NO^−^_2_ to NH^+^_4_ (step 10 and 16). NH^+^_4_ assimilation takes place in the plastid and leads to the formation of glutamine and glutamate (step 11–12 and step 17–18) through glutamine synthetase/glutamate synthase (GS/GOGAT). Nitrate and nitrate reductases are available in all tissues and various isoforms of GS/GOGAT enzymes (GS: EC 6.3.1.2, NADH-GOGAT: EC 1.4.1.3, and ferredoxin (Fd)-GOGAT: EC1.4.7.1) enable these reactions in both photosynthetic and non-photosynthetic tissues.

The histogram for each reaction shows the change in flux distributions caused by the tissue translocation penalties. The flux distribution shapes give information about the sensitivity of the solution space to each constraint. Introduction of an energy cost of transportation greatly affected the probability distributions through many reactions in the network. For instance, the flux distribution for glutamate translocation changed not only in shape, but also in direction (step 14). Under free tissue-translocation (no penalties), the model predicts that nitrate is taken up in roots (sink tissues) but is preferentially assimilated into glutamate in leaves (source tissues). However, under high energy demand for tissue translocation (with penalties), nitrate is preferentially assimilated in roots.

According to the simulations, the translocation constraint is likely to affect many reactions involved in nitrogen metabolism, whereas steps through carbon fixation, photon uptake, starch synthesis/degradation, nitrate uptake and glutamate synthase (in source tissues) are less likely to be affected based on the very similar flux distributions (steps 1–4, 7). Differences in sensitivity of fluxes carried by isoforms of GS/GOGAT enzymes are also highlighted. Our results suggest that the flux through plastidic glutamate synthase in root is likely to be more sensitive to the translocation constraint than its isoform in leaves.

### Coupling analysis in the N uptake and assimilation pathways

Uniform random sampling of the steady-state flux space was used to calculate the correlation coefficient between subsets of fluxes of the N uptake and assimilation pathways under different tissue translocation constraints (Figure 3B). The method also enabled us to identify highly-correlated but not perfectly-correlated reaction subsets. Perfect positive and negative correlations (1.0, −1.0) are shown in red and blue, respectively.

Identification of the correlated reaction sets can aid experimental design. The measurement of any flux in a perfectly correlated reaction set determines the steady-state flux level though all the reactions (Price et al., 2004). For instance, our analysis shows that glutamate translocation between sink and source tissues is perfectly correlated to fluxes through nitrate/nitrite reductases (in leave) and to sucrose translocation, independent of the translocation penalties. Meaning for example that any flux perturbation through the nitrate/nitrite reductases in leave (step 9–10) caused by genetic modifications is likely to affect glutamate (step 14) and sucrose translocation (step 5–6) in between source and sink tissues. The analysis also shows that inefficiency in photon absorption or limited light exposure (step 1), is likely to affect nitrate uptake in roots (step 7), independently of the tissue-translocation constraint.

The translocation penalties affected the correlation of a few reaction sets. For instance, glutamate translocation (step 14) is only perfectly correlated to fluxes through nitrate/nitrite reductases in root (step 15–16) under none or minimum restriction in energy requirements for tissue translocation.

In the absence of experimentally determined cost of active transport, the subsequent studies of the effect of nitrogen source was performed assuming no (or minimal) tissue translocation penalties.

### Flux variability analysis of N contrasts: NO^−^_3_ vs. NH^+^_4_ uptake

Flux variability analysis (FVA) was performed under two nitrogen sources. The predicted uptake rates of nitrate or ammonium are the same during day and night, approximately 7 mmol/g tissue.h under the tested conditions (Table S3, Supplemental Material). This is a direct consequence of the assumptions that (a) growth is constant day and night and (b) there is no nitrogen storage compound to transfer nitrogen between day and night. While this is evidently a gross simplification of metabolism, there is experimental evidence that the enzymes of the NO^−^_3_ and NH^+^_4_ assimilation pathways are active over the diurnal cycle at least in tobacco roots (Stohr and Mack, 2001).

**Figure 4:**
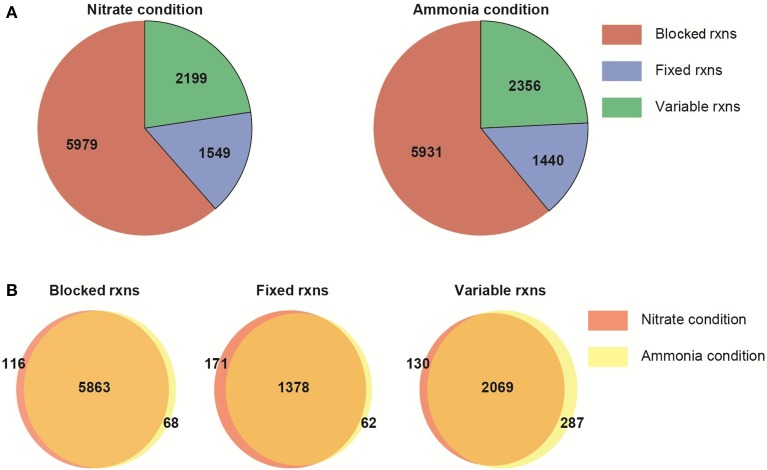
**Flux variability analysis of the multi-tissue metabolic network. (A)** General network characteristics under two nitrogen sources. **(B)** Number of shared and unique reactions under nitrate or ammonia uptake. Blocked reactions: reactions carrying zero flux; fixed rxns: reactions with a fixed, non-zero flux (due to the objective function or the imposition of constraints); variable rxns: reactions with a non-zero flux range.

The metabolic network characteristics are presented for NO^−^_3_ or NH^+^_4_ condition (Figure 4). The network differs only due to the imposition of the nitrogen usage as a constraint. The remaining constraints (i.e., biomass growth rate, biomass composition, reaction with fixed boundaries) are the same in both N conditions. The imposition of the constraints and optimality criterion define the reactions carrying: zero, fixed and variable fluxes (non-zero flux range). Following this preliminary analysis, we then compared the FVA results between the two different nitrogen sources, assuming photon efficiency.

### Nitrate use incurs significantly higher metabolic cost

**Figure 5:**
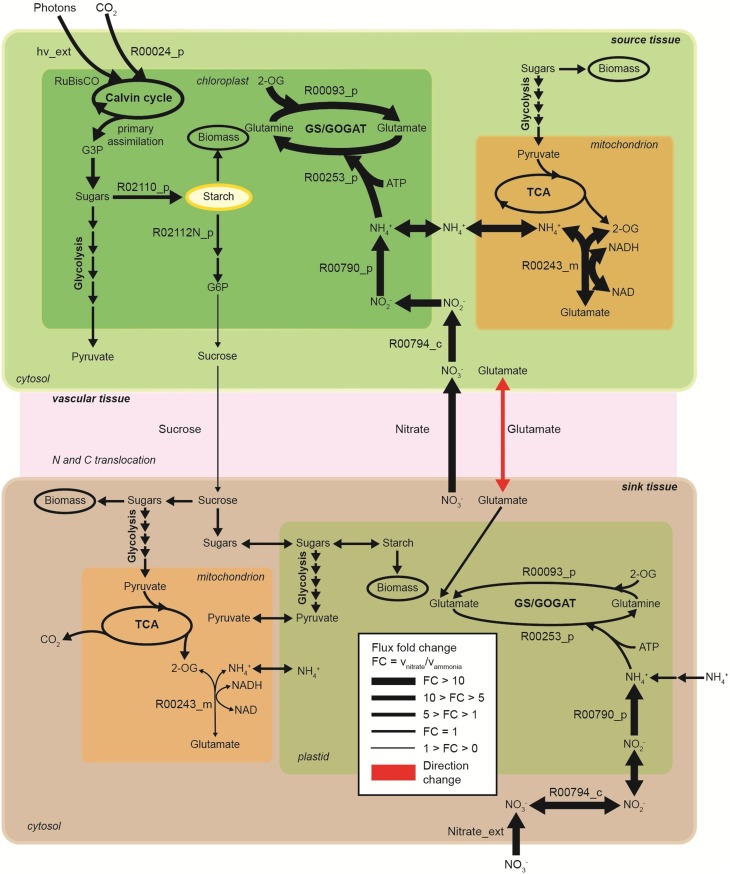
**Metabolic flux contrast during light period in the nitrogen uptake and assimilation pathways across source and sink tissues, under sole nitrate compared to sole ammonia as nitrogen source (no translocation penalty considered)**. Enzymatic step reactions are displayed based on the model reaction IDs. R00794_c, cytosolic nitrate reductase; R00794_p, plastidic nitrite reductase; R00253_p, plastidic glutamine synthase; R00093_p, plastidic glutamate synhtase, R00243_m, mitochondrial glutamate dehydrogenase; R02110_p, starch branching enzyme; R02112N_p, beta-amylase; R00024, ribulose-bisphosphate carboxylase; G3P, glyceraldehyde-3-phosphate; hv_ext, photons uptake. Direction of glutamate translocation: from source tissue to sink tissue under nitrate condition and from sink tissue to source tissue under ammonia condition.

The model shows significant metabolic changes in N uptake and assimilation pathways due to the use of N source (Figure 5). The plant network flexibility does not overcome the fundamentally higher cost of using NO^−^_3_ rather than NH^+^_4_. The model predicts an increase of approximately 17% in photosynthesis and C fixation (measured by photon uptake rate) is required on NO^−^_3_ compared to NH^+^_4_ to sustain the same plant growth. This is a direct result of the cost of nitrate and nitrite reduction.

Consequently, the multi-tissue model predicts flux increases in carbon assimilation and starch accumulation in leaf tissues, in order to sustain the same plant growth rate under NO^−^_3_ metabolism. An increase of approximately 27% in starch accumulation in leaf tissues is required in order to keep the same leave biomass composition (i.e., avoiding any biomass penalties) and to sustain the same plant growth rate. The increase in starch accumulation is achieved by increasing carbon fixation and photons usage. Stored starch in the source tissue during the day is degraded during the night into sugars that are then used to sustain leaf tissue biomass and some are translocated to sustain the sink tissues. Our simulations show no changes in starch degradation during the day. However, flux through starch degradation increases over night in leaf tissues to sustain plant growth (Table S3, Supplemental Material).

Plant growth relies on the efficient and controlled distribution of sucrose and source-to-sink transport of sugars is a major determinant of growth. For most plants, this occurs by loading sucrose into the phloem and transporting it from source tissues to sink tissues, where sucrose in unloaded (Giaquinta, 1977; Truernit and Sauer, 1995; Srivastava et al., 2008). Given constant growth rates during day and night, it is unsurprising that sucrose translocation from source to sink tissues have similar rates over these periods (Supplemental Material, Table S3). However, a decrease in sucrose translocation flux from source to sink tissues is required in order to sustain the same plant growth rate when NO^−^_3_ is the nitrogen source compared to NH^+^_4_. On the other hand, nitrate translocation from sink to source tissue is increased. The model predicts that N is preferentially assimilated into glutamine in leaves, and glutamate translocation from source to sink tissue is increased on NO^−^_3_ compared to NH^+^_4_ usage.

Overall, our model predicts that changes in nitrogen uptake and assimilation pathways are intimately coupled to changes in carbon fixation, photoassimilates and carbon translocation from source to sink tissues. Simulation results support the hypothesis that source-to-sink sucrose can be affected by nitrogen supply and shows that the carbon-nitrogen balance and partitioning are controlled by (i) the supply of assimilates via photosynthesis, (ii) nitrogen source, and (iii) ability of different organs to utilize the available supply.

### Visualizing coupled reaction modules

**Figure 6:**
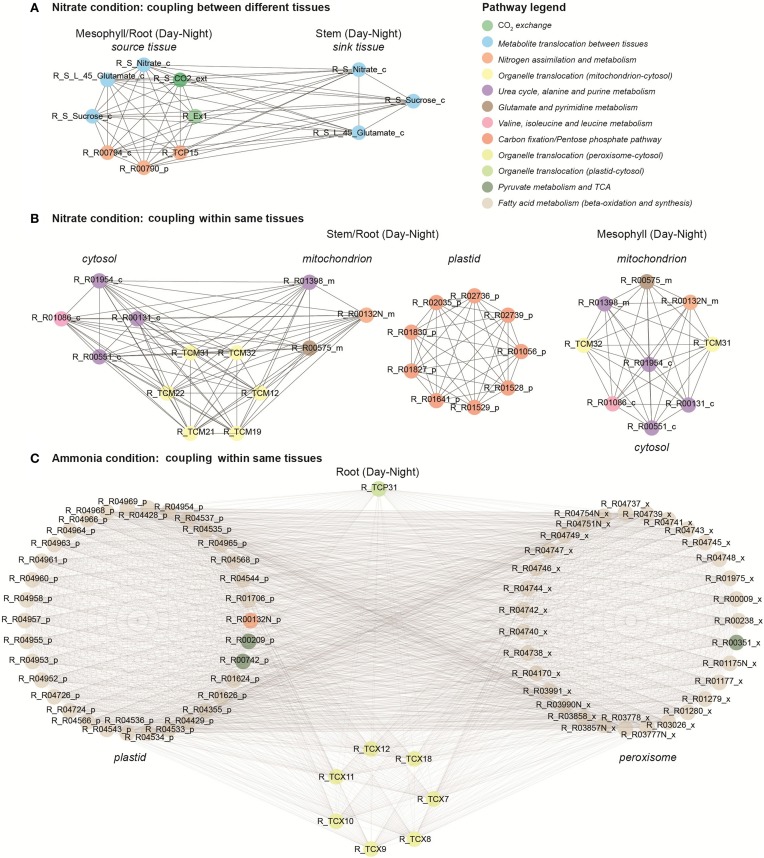
**Coupling analysis under nitrate and ammonia conditions. (A)** Highly coupled reactions in different tissues under nitrate condition. **(B)** Coupled reactions within the same tissue and between different organelles under nitrate condition. **(C)** Coupled reactions within the same tissue and between different organelles under ammonia condition.

Clique analysis has been used previously to visualize the correlation structure of *A. thaliana* metabolomics data and to obtain further information about metabolite relationships (Kose et al., 2001). Here, we used clique analysis to visualize the largest sets of fully connected reaction modules for different conditions and different correlation cut-offs (ρ_cut−off_) under optimal photon uptake (Supplemental Material, Table S4). Figure 6 presents the largest sets of fully connected reaction modules (i.e., maximal cliques) under different N sources using a high correlation threshold (ρ_cut−off_ = 0.95). Each coupled reaction pair is represented as two vertices connected by an edge. Coupling analysis enabled us to identify and visualize highly coupled reactions from different tissues, organelles and day periods under different conditions. For instance, two reaction modules coupled between source and sink tissues under nitrate condition (Figure 6A). The nodes shown in blue are “tissue linkers” and represent the C and N species translocated in between source and sink tissues (e.g., sucrose, nitrate and glutamate). These transporters are highly coupled to step reactions of N and C assimilation in source tissues. Similarly, we observed clusters of highly coupled reactions between different organelles within stem and root tissues under nitrate condition (Figure 6B). The first cluster represents highly coupled reactions between cytosol and mitochondrion. The second cluster displays highly coupled reactions of the pentose phosphate pathway and C fixation in plastids, whereas the third cluster shows highly correlated reactions in mitochondrion. Under ammonia condition, the maximal clique was found in root tissue (Figure 6C). This cluster includes reactions involved in fatty acid synthesis (in plastids) and beta-oxidation (in peroxisome) pathways. Interestingly, we found that most of these step reactions are blocked (zero flux) under the nitrate condition. These are only a few examples of how non-trivial correlations can be obtained from a topological analysis of the multi-tissue network.

## Concluding remarks

Efforts toward multi-tissue and ultimately whole-plant models will form an important component of genome scale computational models of plant growth and development and are likely to play a major role in efforts to improve crop yield and quality. Here, we have presented a flexible multi-tissue modeling framework to study whole plant resource allocation over the diurnal cycle, coupling both spatial (via cell/tissue interfaces) and temporal (across the diurnal cycle) processes.

This approach may generally be coupled to extensive omics data sets in a two way interaction where data narrows the solution space and the network model enable us to gain biologically meaningful insights into complex networks and system level interactions.

In the current study, we did not constrain the tissue models using gene expression data for leaf, stem or root tissues. Instead, we used the minimum set of constraints for key biochemical reactions that distinguish phototrophic (leaf) and heterotrophic (stem and root) tissues. This enabled us to explore the optimum distribution of metabolic fluxes in the whole plant system, assuming that plant metabolism (e.g., kinetics and regulatory factors) has evolved to become efficient at utilizing photons for growth.

With minimal constraints, the solution space remains large. Using random sampling, however, we were still able to unravel network-wide effects of (a) imposing energy penalties to account for active transport and (b) using NO^−^_3_ rather than NH^+^_4_ as nitrogen source. Identification of highly correlated reactions sets enabled visualization of key pathways linking metabolic reactions from different tissues, organelles and day periods under different conditions. The examples of *in silico* flux predictions illustrate the potential of this framework to interrogate plant metabolism at the multi-tissue level.

### Conflict of interest statement

The authors declare that the research was conducted in the absence of any commercial or financial relationships that could be construed as a potential conflict of interest.
