# Supplementary material for: A multi-tissue genome-scale metabolic modeling framework for the analysis of whole plant systems
Source: Front Plant Sci. 2015 Jan 22;6:4. doi: 10.3389/fpls.2015.00004 (PMC4302846; doi:10.3389/fpls.2015.00004)
Supplement: Supplementary file 4 [file Table4.DOCX]

*Clique analysis*

The network analyses were performed under four contrasts: (i) nitrate or (ii) ammonia usage, (iii) no penalties assuming free C/N tissue translocation for the non-free energetic process of translocation (iv) with penalties for tissue trasnlocation. The resulting number of cliques was altered by varying the correlation threshold (Table1).

Table S4. Coupling analysis with and without translocation penalty for different correlation cut-offs (*ρ*_cut-off_) under optimal photon uptake

| *ρ*_cut-off_ | Ammonia condition with penalty | | | | Nitrate condition without penalty | | | |
| --- | --- | --- | --- | --- | --- | --- | --- | --- |
|  | Coupled reaction pairs | Coupled reaction pairs in different tissues | Cliques | Cliques involving different tissues | Coupled reaction pairs | Coupled reaction pairs in different tissues | Cliques | Cliques involving different tissues |
| 0.95 | 7903 | 12 | 648 | 4 | 2880 | 108 | 645 | 8 |
| 0.90 | 8215 | 18 | 660 | 4 | 3133 | 120 | 653 | 8 |
| 0.85 | 8457 | 18 | 675 | 4 | 3408 | 128 | 670 | 9 |
| 0.80 | 8651 | 18 | 684 | 4 | 3662 | 140 | 684 | 9 |
| 0.75 | 8893 | 19 | 701 | 5 | 4125 | 328 | 705 | 13 |
| 0.70 | 9312 | 77 | 729 | 16 | 4624 | 372 | 740 | 14 |
| 0.65 | 10172 | 286 | 765 | 52 | 5279 | 597 | 774 | 40 |
| 0.60 | 11786 | 980 | 883 | 159 | 6431 | 1065 | 886 | 117 |
