# Supplementary material for: A multi-tissue genome-scale metabolic modeling framework for the analysis of whole plant systems
Source: Front Plant Sci. 2015 Jan 22;6:4. doi: 10.3389/fpls.2015.00004 (PMC4302846; doi:10.3389/fpls.2015.00004)
Supplement: Supplementary file 5 [file DataSheet1.ZIP › multi-tissue framework/read me.docx]

**Directions to use the multi-tissue framework (for windows)**

What do you need installed in your computer?

-Matlab v7.10.0 (R2010a) or superior version

-Cplex optimizer: <http://www-01.ibm.com/software/commerce/optimization/cplex-optimizer/>

or Gurobi optimizer: <http://www.gurobi.com/download/gurobi-optimizer>

**Input files:**

1. Model (sbml)
2. MyModelSpect (.xlsx)

**m.scripts released:**

a) step1_readSources.m

b) step2_loadMultiTissues.m

c) step2a_writeModelData2XLS.m

d) step3_fluxSim.m

e) runall.m

**Get start:**

1. Save the metabolic reconstruction (sbml format) in the multi-tissue framework folder. In this package, AraGEM was saved as ‘model.xml’.
2. Enter the parameters in ‘myModelSpecs’ file. This is a xlsx file with 6 sheets named:

sbml, compartment, metConnections, biomassDrain, lbub, and objFxn.

1.1) ‘sbml’ sheet: define the name of the metabolic reconstruction. E.g.: model.xml

1.2) ‘compartments’ sheet: enter tissues, spatial tags, temporal tags, tissue weight and day/night hours.

1.3) ‘metConnections’ sheet: Define the metabolites translocated in between the tissues in column B. Define tissue for each metabolite in column C. Tag the commonPool. Example, MSD tag referrers to metabolites translocated between leaf (mesophyll) and stem during the day. See abbreviations in the sheet.

1.4) biomassDrain sheet: enter the biomass fractions for each tissue

1.5) lbub sheet: enter reaction constraints

1.6) objFxn sheet: enter objective function

Save ‘myModelSpecs’ file.

1. Open matlab
   1. Direct current folder to the multi-tissue framework folder
   2. Add pathway to Cplex optimizer in start up.m file. Example:

addpath 'C:\multi-tissue framework\x64_win64'

- 1. Flux simulator: Run the m. script named runall.m. The multi-tissue model is generated in the screen and flux solution is solved in few seconds.

source read done

original model...

no of mets: 1782

no of rxns: 1607

tissue block addition...

1 Mesophyll M D 1 12

2 Stem S D 2 12

3 Root R D 3 12

4 Mesophyll M N 1 12

5 Stem S N 2 12

6 Root R N 3 12

no of mets: 10692

no of rxns: 9642

biomass drain reactions addition...

M D

S D

R D

M N

S N

R N

metabolite connections addition...

1 S_Sucrose_c M D MSD 1 1

2 S_Sucrose_c S D MSD -1 2

3 S_Sucrose_c S D SRD 1 1

4 S_Sucrose_c R D SRD -1 3

5 S_H2O_c M D MSD -1 0

6 S_H2O_c S D MSD 1 0

7 S_H2O_c S D SRD -1 0

8 S_H2O_c R D SRD 1 0

9 S_Sulfate_c M D MSD -1 3

10 S_Sulfate_c S D MSD 1 1

11 S_Sulfate_c S D SRD -1 2

12 S_Sulfate_c R D SRD 1 1

13 S_Orthophosphate_c M D MSD -1 0

14 S_Orthophosphate_c S D MSD 1 3

15 S_Orthophosphate_c S D SRD -1 0

16 S_Orthophosphate_c R D SRD 1 2

17 S_L_45_Glutamate_c M D MSD -1 3

18 S_L_45_Glutamate_c S D MSD 1 1

19 S_L_45_Glutamate_c S D SRD -1 2

20 S_L_45_Glutamate_c R D SRD 1 1

21 S_Nitrate_c M D MSD -1 3

22 S_Nitrate_c S D MSD 1 1

23 S_Nitrate_c S D SRD -1 2

24 S_Nitrate_c R D SRD 1 1

25 S_Sucrose_c M N MSN 1 1

26 S_Sucrose_c S N MSN -1 3

27 S_Sucrose_c S N SRN 1 1

28 S_Sucrose_c R N SRN -1 2

29 S_H2O_c M N MSN -1 0

30 S_H2O_c S N MSN 1 0

31 S_H2O_c S N SRN -1 0

32 S_H2O_c R N SRN 1 0

33 S_Sulfate_c M N MSN -1 3

34 S_Sulfate_c S N MSN 1 1

35 S_Sulfate_c S N SRN -1 2

36 S_Sulfate_c R N SRN 1 1

37 S_Orthophosphate_c M N MSN -1 0

38 S_Orthophosphate_c S N MSN 1 3

39 S_Orthophosphate_c S N SRN -1 0

40 S_Orthophosphate_c R N SRN 1 2

41 S_L_45_Glutamate_c M N MSN -1 3

42 S_L_45_Glutamate_c S N MSN 1 1

43 S_L_45_Glutamate_c S N SRN -1 2

44 S_L_45_Glutamate_c R N SRN 1 1

45 S_Nitrate_c M N MSN -1 3

46 S_Nitrate_c S N MSN 1 1

47 S_Nitrate_c S N SRN -1 2

48 S_Nitrate_c R N SRN 1 1

45 S_Starch_biomass M D DNLINK1 1 0

46 S_Starch_biomass M N DNLINK1 -1 0

47 S_CO2_ext M D BOUNDARY 0 0

48 S_H2O_ext M D BOUNDARY 1 0

49 S_Oxygen_ext M D BOUNDARY 0 0

50 S_hv_ext M D BOUNDARY -1 0

51 S_CO2_ext S D BOUNDARY 0 0

52 S_H2O_ext S D BOUNDARY 1 0

53 S_Oxygen_ext S D BOUNDARY 0 0

54 S_Nitrate_ext R D BOUNDARY -1 0

55 S_NH3_ext R D BOUNDARY -1 0

56 S_Sulfate_ext R D BOUNDARY -1 0

57 S_H2O_ext R D BOUNDARY -1 0

58 S_CO2_ext R D BOUNDARY 0 0

59 S_Orthophosphate_ext R D BOUNDARY -1 0

60 S_Oxygen_ext R D BOUNDARY 0 0

61 S_Hydrogen_sulfide_ext R D BOUNDARY -1 0

62 S_CO2_ext M N BOUNDARY 0 0

63 S_H2O_ext M N BOUNDARY 1 0

64 S_Oxygen_ext M N BOUNDARY 0 0

65 S_CO2_ext S N BOUNDARY 0 0

66 S_H2O_ext S N BOUNDARY 1 0

67 S_Oxygen_ext S N BOUNDARY 0 0

68 S_Nitrate_ext R N BOUNDARY -1 0

69 S_NH3_ext R N BOUNDARY -1 0

70 S_Sulfate_ext R N BOUNDARY -1 0

71 S_H2O_ext R N BOUNDARY -1 0

72 S_CO2_ext R N BOUNDARY 0 0

73 S_Orthophosphate_ext R N BOUNDARY -1 0

74 S_Oxygen_ext R N BOUNDARY 0 0

75 S_Hydrogen_sulfide_ext R N BOUNDARY -1 0

specifying objective functions

1 met S_hv_ext M D BOUNDARY -1

specifying boundary constraints

1 bio M, S, R D, N 1 1

2 rxn R_R00472_x M,S,R D,N 0 10000

3 rxn R_R03140_p M D 150 10000

4 rxn R_Ex5 R D,N -0 0

5 rxn R_R00024_p S,R D,N 0 0

6 rxn R_R00253_p M,S,R D,N 0 10000

7 rxn R_R00253_m M,S,R D,N 0 10000

8 rxn R_R00086_c M,S,R D,N 0 10000

model generated

final model...

no of mets: 10732

no of rxns: 9727

rxn write successful

internal metabolites write successful

external metabolites write successful

fval: 5733.116

exitflag: 1

**Output files are generated in the multi-tissue folder:**

a) multiTissuesModel.xslx

b) x.xslx (flux output)
